# Comparative Acquisition, Transmission, and Retention of Distinct Grapevine Red Blotch Virus Isolates in Relation to the Genotype and Sex of *Spissistilus festinus*, the Treehopper Vector

**DOI:** 10.3390/v17091274

**Published:** 2025-09-20

**Authors:** Victoria J. Hoyle, Anna O. Wunsch, Heather McLane, Scottie Browning, Madison T. Flasco, Elizabeth J. Cieniewicz, Marc Fuchs

**Affiliations:** 1 Section of Plant Pathology and Plant-Microbe Biology, School of Integrative Plant Science, Cornell University, Geneva, NY 14456, USA; aw838@cornell.edu (A.O.W.); hlm9@cornell.edu (H.M.); mf13@cornell.edu (M.F.); 2 Department of Plant Pathology and Crop Physiology, Louisiana State University Agricultural Center, Baton Rouge, LA 70803, USA; mflasco@agcenter.lsu.edu; 3 Department of Plant and Environmental Sciences, Clemson University, Clemson, SC 29634, USA; ecienie@clemson.edu

**Keywords:** acquisition, grapevine red blotch virus, retention, *Spissistilus festinus*, transmission

## Abstract

Grapevine red blotch virus (GRBV), the causal agent of red blotch disease of grapevines, is transmitted by *Spissistilus festinus*, the threecornered alfalfa hopper. Isolates of GRBV belong to two phylogenetic clades (I and II) and *S. festinus* is a dimorphic insect, with two genotypes found in the western (California, CA) and the southeastern (SE) regions of the United States. The transmission of GRBV by *S. festinus* is circulative and nonpropagative, yet some parameters of transmission remain to be characterized. Here, we compared the acquisition, transmission, and retention of GRBV isolates from phylogenetic clades I and II by *S. festinus* males and females of the two genotypes. Results indicated that the SE genotype acquired GRBV more efficiently (72.5%, 29/40) than the CA genotype (22.5%, 18/80), with differences in acquisition observed between males (32.5%, 26/80) and females (52.5%, 21/40) of the two *S. festinus* genotypes and between GRBV isolates of phylogenetic clades I (29%, 23/80) and II (60%, 24/40). Following acquisition, both *S. festinus* genotypes and sexes retained GRBV isolates of phylogenetic clades I and II for at least 60 days without access to an infected plant. For transmission, the GRBV isolate of phylogenetic clade II was more efficiently transmitted by the SE genotype (54%, 13/24) than the CA genotype (17%, 4/24) and SE females (75%, 12/16) were significantly more efficient transmitters of GRBV than CA females (19%, 3/16). Together, our findings revealed that *S. festinus* genotype, sex, and virus isolate influence GRBV acquisition and transmission but not retention. This research addressed important knowledge gaps in *S. festinus*-mediated transmission of GRBV that are essential for advancing red blotch disease epidemiology and developing appropriate disease management responses.

## 1. Introduction

Red blotch is a viral disease that threatens the wine and grape industries in North America [1]. The estimated economic impact in the United States (U.S.) is up to $68,548 per hectare over the expected 25-year lifespan of a ‘Cabernet Sauvignon’ vineyard [2]. Red blotch disease is caused by grapevine red blotch virus (GRBV, species *Grablovirus vitis*, genus *Grablovirus*, family *Geminiviridae*) [3]. The only natural hosts of GRBV are *Vitis* species, including wine grapes (*V. vinifera*), table grapes, rootstocks, interspecific hybrids, muscadines, and free-living vines in southern Oregon (*V. riparia*) and northern California (*V. californica* and its hybrids) [1]. Outside North America, GRBV has been reported in vineyards in South Korea, Mexico, Argentina, India, and Iran. It has also been described in *Vitis* germplasm repositories in the U.S., Switzerland, Italy, France, and Australia [1].

GRBV has a circular, single-stranded DNA genome with seven putative open reading frames (ORFs), three in the complementary orientation (C1–C3) and four in the viral orientation (V0–V3) [1]. The GRBV genome is larger than most geminiviruses [4], with 3206 bp. Protein V1 is the predicted coat protein and proteins V2 and V3 are likely movement proteins based on their subcellular localization. Proteins V2 and C2 are suppressors of RNA silencing and the Rep-associated protein results from a splicing event fusing the C1 and C2 ORFs. No functions are yet ascribed to proteins V0 and C3 [1].

Two major phylogenetic lineages (I and II) of GRBV sequences have been identified with up to 8.5% nucleotide divergence. GRBV variants of phylogenetic clade II are the most prevalent in vineyards [4]. Representative sequences from both phylogenetic clades were used for engineering GRBV infectious clones to fulfill Koch’s postulates [3] and to inoculate plants for vector-mediated transmission assays [5,6,7,8]. Of note, following *Agrobacterium tumefaciens*-mediated delivery of virus infectious clones, snap bean (*Phaseolus vulgaris*) can be used as a pseudo-systemic herbaceous host of GRBV for facilitating transmission biology studies [5,6,7,8,9].

GRBV is transmitted by the treehopper, *Spissistilus festinus* [Say, 1830] (Hemiptera: Membracidae), the threecornered alfalfa hopper, in a circulative, nonpropagative manner [1,5,9]. This means that the virus needs to transit through the body of the treehopper vector to reach the salivary glands for transmission to occur [5]. Virus acquisition by *S. festinus* individuals can therefore be determined by ascertaining the presence of GRBV in the salivary glands following feeding on an infected host and then on a non-host to clear the mouth parts and digestive tract. Furthermore, in analogy with other geminiviruses, transmission of GRBV by *S. festinus* is transstadial but not transovarial [4,5,6,7].

GRBV is transmitted by *S. festinus* from and to wine grapes, from and to free-living vines, and between wine grapes and free-living vines [6]. Previous work carried out in vineyards of northern California revealed more *S. festinus* males (17%) than females (8%) testing positive for GRBV [10], suggesting a greater potential for GRBV acquisition by *S. festinus* males. Similarly, analyses of the flight capacity of *S. festinus* indicated significantly farther flights of males compared to females (570.22 m vs. 239.57 m), supporting a potentially greater capacity of males to spread GRBV in vineyards [11]. Recent work in the laboratory confirmed higher transmission rates of GRBV by *S. festinus* males (17%) than females (4%) when allowed to move freely within experimental arenas [7].

Two genotypes of *S. festinus* have been identified in the U.S., one from the southeastern U.S. (SE) and the other from the western U.S. (California, CA, USA). These genotypes were identified based on sequence divergence in the barcoding genes ITS2 and mt-COI [12]. Transmission assays have demonstrated that *S. festinus* specimens from the SE genotype are more efficient vectors of a GRBV variant belonging to phylogenetic clade I (89%) in comparison to specimens from the CA genotype (50%) [13]. GRBV variants from phylogenetic clade I and clade II are spreading in northern California vineyards [14,15,16], but potential differences in their transmission rates, particularly by the two *S. festinus* genotypes, are not known.

Despite considerable progress on transmission biology, a comprehensive understanding of how GRBV acquisition and transmission might be influenced by *S. festinus* genotype and sex, as well as by viral variants, is lacking. Similarly, no information is available on the retention of GRBV by *S. festinus*, although by analogy with other geminiviruses [4], the virus is anticipated to be retained for the lifespan of the treehopper, or approximately three months [17]. The aim of our study was to investigate rates of GRBV acquisition, transmission, and retention in relation to treehopper vector characteristics and virus diversity. Based on our previous observations [7,10,13], we hypothesized that specimens of the *S. festinus* SE genotype are more efficient vectors of GRBV compared to specimens of the CA genotype, particularly males from either genotype compared to females. We further hypothesized that viral genetic variants of the two phylogenetic clades are similarly transmitted by *S. festinus*, regardless of the genotype and sex of the treehopper vector. Here, we summarize our comparative acquisition, transmission, and retention assays of distinct GRBV variants using males and females of the two *S. festinus* genotypes.

## 2. Materials and Methods

### 2.1. Plant Material

Snap bean (*Phaseolus vulgaris*, cv. ‘Hystyle’), a pseudo-systemic host of GRBV [5,6,7,8,9], was used for virus acquisition, transmission, and retention assays. Alfalfa (*Medicago sativa*, Galaxy 100 blend), a non-host of GRBV, was used for *S. festinus* gut clearing in acquisition and retention assays [5].

Seeds of snap bean and alfalfa were sown and transplanted in LM-3 soil mix (Lambert, Rivière-Ouelle, Québec, QC, Canada). Seedlings were grown in a greenhouse at 26 ± 4 °C and 70% relative humidity with a 14:10 h light/dark photoperiod at Cornell AgriTech, Geneva, NY, USA.

### 2.2. Spissistilus festinus Colonies

Populations of the SE and CA genotypes of *S. festinus* were reared separately on *P. vulgaris* cv. ‘Hystyle’ and maintained in controlled environment chambers at 25 °C with a 14:10 h light/dark photoperiod and 70% relative humidity at Cornell AgriTech, Geneva, NY, USA [18]. The SE colony was derived from *S. festinus* caught from garden bean plants in Pickens County, SC, USA in 2021. The CA colony was derived from *S. festinus* caught in alfalfa fields in Fresno, Yolo, and San Joaquin Counties, CA, USA, between 2015 and 2019. Genotypes were previously confirmed using diagnostic PCR assays and Sanger sequencing [12,13].

### 2.3. Inoculation of Snap Bean Plants with GRBV Infectious Clones via Agro-Pricking

Snap bean plants were inoculated by pinpricking with sterile dissecting needles coated in a solid culture of *Agrobacterium tumefaciens* strain C58C1 containing an infectious clone of either GRBV isolate NY175 (clade I) or NY358 (clade II), as previously described [8]. Additional snap bean plants were mock inoculated by pinpricking with non-coated sterile dissecting needles. Inoculated plants were maintained for one week in a greenhouse at 26 ± 4 °C and 70% relative humidity with a 14:10 h light/dark photoperiod at Cornell AgriTech, Geneva, NY, USA [8] prior to the acquisition, transmission, and retention experiments.

### 2.4. Acquisition Assays of GRBV by Spissistilus festinus

**Figure 1:**
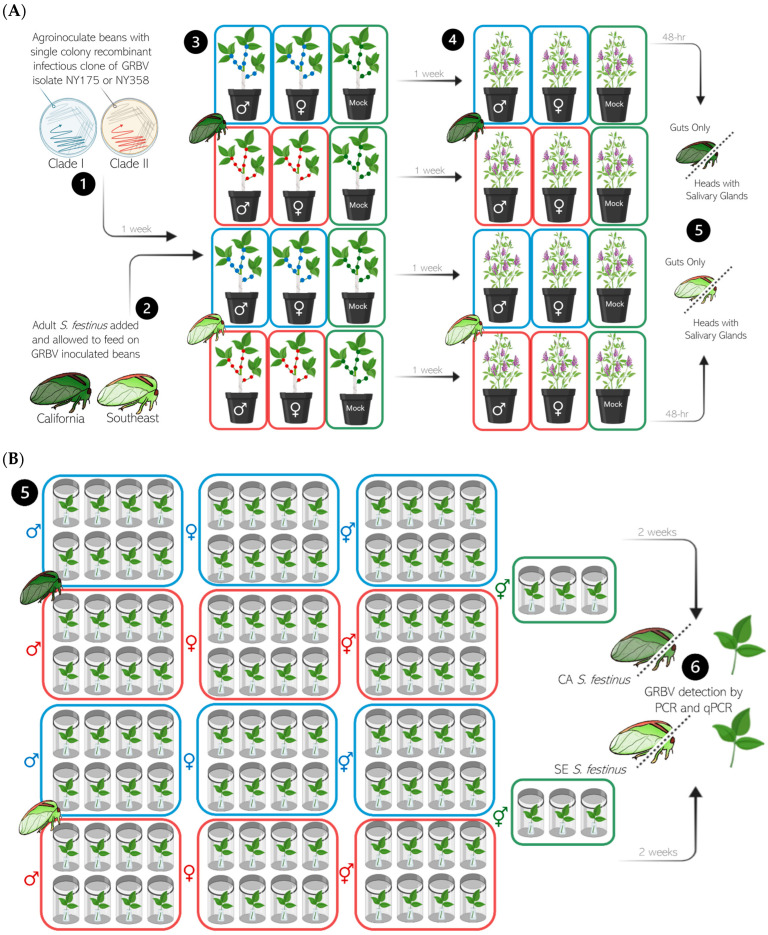
(**A**) Schematic representation of the acquisition assays of grapevine red blotch virus (GRBV) isolates from phylogenetic clades I and II by *Spissistilus festinus*. Briefly, (**1**) snap bean plants were inoculated by pinpricking petioles of the trifoliates with solid cultures of *Agrobacterium tumefaciens* strain C58C1 cells carrying infectious clones of a GRBV isolate from phylogenetic clade I (NY175, blue) or clade II (NY358, red) and grown in a greenhouse for 7 days, (**2**) males or females of the Southeast and California *S. festinus* genotypes were transferred to infected snap bean plants alongside mock-inoculated plants (green), and (**3**) allowed to feed separately for 7 days. After a 48 h gut clearing period on alfalfa (**4**), individual male or female treehoppers were captured and dissected under an Olympus SZX16 stereo microscope to isolate heads with salivary glands and guts for GRBV testing by multiplex PCR. The artwork was produced using the program BioRender (Toronto, ON, Canada) and artwork by Dr. Brandon Roy, Cornell University. (**B**). Schematic representation of the transmission assays of GRBV isolates from phylogenetic clades I (blue) and II (red) by *Spissistilus festinus*. Following acquisition (Figure 1A) (**5**), males only, females only or a combination of males and females of the Southeast and California *S. festinus* genotypes were separately transferred to uninoculated trifoliates of snap bean plants alongside trifoliates of mock-inoculated plants (green) and allowed to feed for 7 days. Then (**6**), individual trifoliates were collected and individual male and female insects were captured and dissected under an Olympus SZX16 stereo microscope to isolate heads with salivary glands and guts for GRBV testing by multiplex PCR. The artwork was produced using the program BioRender (Toronto, ON, Canada) and artwork by Dr. Brandon Roy, Cornell University.

Populations of 125–150 newly emerged adult *S. festinus* of each sex and genotype were transferred from the colonies and placed on wrapped snap bean plants inoculated with each GRBV isolate (approximately 50 individuals per plant), contained in BugDorm 6E610 insect rearing cages (MegaView Science, Taichung, Taiwan) and allowed to feed for a one-week acquisition access period (AAP) as previously described [8]. Then, *S. festinus* were transferred onto alfalfa, a nonhost of GRBV [5], for a 48 h gut clearing period (Figure 1A). Finally, individual *S. festinus* were dissected under an Olympus SZX16 stereo microscope (Evident Scientific, Waltham, MA, USA) to isolate guts and heads with salivary glands for GRBV testing by multiplex PCR to verify virus acquisition [5,8,9].

### 2.5. Transmission Assays of GRBV by Spissistilus festinus

Following a one-week AAP and a 48 h gut clearing on alfalfa, cohorts of six *S. festinus* from each treatment were transferred to 2- to 3-week-old non-infected, detached snap bean trifoliates placed in 20 mL glass vials filled with water and sealed with parafilm, within clear polypropylene 950 mL containers (cat. no. PK32T, Fabri-Kal, Kalamazoo, MI, USA) sealed with a polypropylene donut lid carrying a nylon screen [8] (Figure 1B). Insects were maintained on the trifoliates for a two-week inoculation access period (IAP). Eight replicates were used per treatment.

Following the IAP, *S. festinus* individuals were collected using a D-cell powered aspirator (Gemplers, Janesville, WI, USA) and stored at –80 °C for GRBV testing following dissections of heads with salivary glands and guts to be tested separately. Snap bean trifoliates were collected and processed to separate petioles, petiole–leaf junctions, and midveins [8]. Approximately 100 mg of plant tissue from each tissue type was stored at –20 °C in 2 mL collection tubes containing two sterile 4.5 mm steel ball bearings for GRBV testing.

### 2.6. Retention of GRBV in Spissistilus festinus

**Figure 2:**
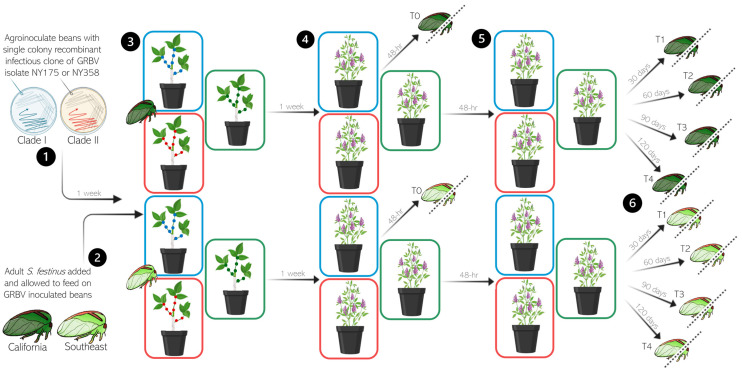
Schematic representation of the retention assays of grapevine red blotch virus (GRBV) isolates from phylogenetic clades I (blue) and II (red) by *Spissistilus festinus.* Steps (**1**–**3**) were conducted as described in Figure 1A, then (**4**) *S. festinus* were moved from snap beans to alfalfa for a 48 h gut clearing period. After the 48 h gut clearing period on alfalfa, individual male and female insects were collected as the T0 timepoint. (**5**) Remaining *S. festinus* were transferred to new alfalfa plants. (**6**) At monthly intervals of 30 (T1), 60 (T2), 90 (T3) and 120 (T4) days following virus acquisition, insects were captured and dissected under an Olympus SZX16 stereo microscope to isolate heads with salivary glands and guts for GRBV testing by multiplex PCR. The artwork was produced using the program BioRender (Toronto, ON, Canada) and artwork by Dr. Brandon Roy, Cornell University.

Populations of 300 newly emerged adult *S. festinus* of each genotype were transferred from the colonies and placed on GRBV-inoculated snap bean plants (75 individuals per plant), contained in BugDorm 6E610 insect rearing cages, and allowed to feed for one week, as previously described [8]. After this one-week AAP, followed by a 48 h gut clearing period on alfalfa, insects were transferred to uninoculated alfalfa plants (75 individuals per plant), contained in BugDorm 6E610 insect rearing cages, and allowed to feed for up to 120 days (Figure 2). Throughout the duration of the experiment, insects were transferred to new alfalfa plants in fresh insect rearing cages and mortality was assessed every 30 days (Supplementary Figure S1).

Subsets of insects from each treatment were collected at 0, 30, 60, 90 and 120 days following the gut clearing period and stored at −80 °C, prior to GRBV testing (Figure 2). *S. festinus* were dissected under an Olympus SZX16 stereo microscope (Evident Scientific, Waltham, MA, USA) to isolate heads with salivary glands and guts for GRBV testing of the two tissues by multiplex PCR and quantitative PCR (qPCR) [8].

### 2.7. Detection of GRBV in Snap Bean Tissue by Multiplex PCR, qPCR, or RCA

Nucleic acids were isolated from snap bean using the MagMAX™ Viral Isolation Kit (ThermoFisher Scientific, Waltham, MA, USA) with the KingFisher™ Flex automated extraction instrument (ThermoFisher Scientific, Waltham, MA, USA) following the manufacturer’s guidelines. GRBV presence in snap bean trifoliates was assessed by multiplex PCR using primer pairs targeting the ORFs encoding the predicted coat protein (CP) and replication-associated protein (RepA) [19], alongside primers targeting the plant 16S housekeeping gene [8,19]. Negative controls included sterile water and nucleic acids isolated from healthy excised bean trifoliates. A snap bean trifoliate was considered infected with GRBV if at least one of the three tissue types, e.g., petioles, petiole–leaf junctions, and midveins, tested positive for the virus in multiplex PCR.

In a few selected snap bean samples for which multiplex PCR outputs were inconclusive, the presence of GRBV was determined by qPCR or rolling circle amplification (RCA). For qPCR, nucleic acid preparations were tested in triplicate (three technical replicates per sample) on a Bio-Rad C1000 Touch thermocycler (Bio-Rad, Hercules, CA, USA) using SYBR Green chemistry (iTaq Universal SYBR Green Supermix; Bio-Rad, Hercules, CA, USA) with primers pREP3v and pREP4v targeting RepA [20], along with primer pair T197 targeting a plant housekeeping gene encoding a guanine nucleotide-binding protein subunit [21], as previously described [8]. RCA was performed using the phi29 DNA polymerase followed by a restriction digestion of the double-stranded DNA product with *PstI* and *Kpn I* [19]. Negative controls for qPCR and RCA included sterile water and nucleic acids isolated from healthy excised bean trifoliates.

### 2.8. Detection of GRBV in Spissistilus festinus Tissue by PCR and qPCR

Genomic DNA was isolated from intact *S. festinus* and dissected heads with salivary glands and dissected guts using the MagMAX™-96 DNA Multi-Sample Kit (ThermoFisher Scientific, Waltham, MA, USA) with the KingFisher™ Flex automated extraction instrument (ThermoFisher Scientific, Waltham, MA, USA) following the manufacturer’s guidelines. GRBV presence in *S. festinus* was initially assessed by multiplex PCR using the aforementioned primer pairs targeting ORFs encoding the CP and RepA [19], along with *S. festinus* primers Sf18Sfor and Sf18Srev targeting a fragment of the 18S gene [8,9]. Negative controls in PCR included sterile water and nucleic acids isolated from *S. festinus* from the colony maintained on GRBV-negative snap bean plants.

Detection of GRBV in *S. festinus* by qPCR utilized the pREP3v and pREP4v primers targeting RepA [20], along with *S. festinus* primers Sf18Sfor and Sf18Srev [8]. Negative controls in qPCR included nucleic acids isolated from *S. festinus* from the colony maintained on healthy snap bean plants. Relative GRBV titer in dissected heads with salivary glands of individual specimens was calculated using the 2^–ΔΔCt^ method [22] with respect to expression of the *S. festinus* 18S housekeeping gene and mock-inoculated *P. vulgaris* tissue.

### 2.9. Statistical Analysis

Statistical analyses were performed using R statistical software v4.5.0 in RStudio v2025.05.1+513 [23,24]. Binomial logistic regression analyses were conducted to evaluate the significance of differences in GRBV acquisition, transmission, and retention rates by *S. festinus* genotypes (SE and CA), sex (male and female), and virus isolates (NY175 and NY358) using the ‘lme4’ package (v1.1-37) and the ‘emmeans’ package (v1.11.1) in R [25,26]. In some instances, Fisher’s exact tests were also conducted using the ‘stats’ package (v4.5.0) in R [23]. Analysis of variance tests were conducted to evaluate the significance of differences in GRBV titer by *S. festinus* genotypes, sex, and virus isolates using the ‘stats’ package (v4.5.0) in R [23]. Tukey’s Honest Significant Difference tests were conducted to assess the significance of differences between group means using the ‘stats’ package (v4.5.0) in R [23]. Relative GRBV titer values above or below 1.5 times the interquartile range were considered outliers and excluded from analysis. Plots were generated using the ‘ggplot2’ (v3.5.2) and ‘ggtext’ (v0.1.2) packages in R [27,28]. Significant differences were identified with *p*-values < 0.05.

## 3. Results

### 3.1. Acquisition of GRBV by Spissistilus festinus

Prior to investigating GRBV acquisition by *S. festinus*, the relative GRBV titer in every snap bean plant that was inoculated with one of the two virus isolates was determined by qPCR at the completion of AAP. Results showed no significant difference in the relative GRBV titer (Ct values of 22.47–27.59) between virus isolates (*p* = 0.517,Tukey’s Honestly Significant Difference), cohorts of inoculated plants fed on by the two *S. festinus* genotypes (*p* = 0.455, Tukey’s Honestly Significant Difference) or sexes (*p* = 0.904, Tukey’s Honestly Significant Difference). These data suggested that the treehoppers had access to similar amounts of GRBV by feeding on infected plant tissue.

**Figure 3:**
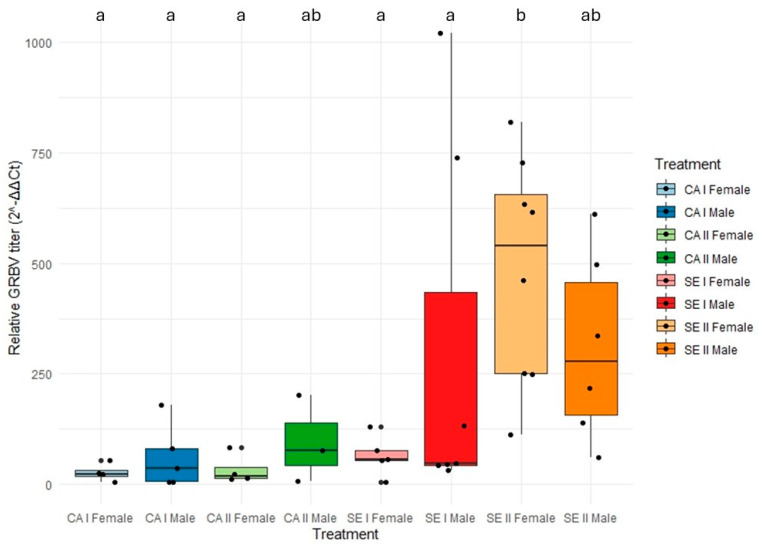
Relative grapevine red blotch virus (GRBV) titer in dissected heads with salivary glands of *Spissistilus festinus* individuals expressed as 2^–ΔΔCt^ values. Treatments comprise cohorts of male-only or female-only *S. festinus* of the California (CA) or Southeast (SE) genotypes following an acquisition access period (AAP) on *Phaseolus vulgaris* (snap bean) plants inoculated with GRBV isolate NY175 (phylogenetic clade I) or NY358 (phylogenetic clade II). Lowercase letters above each treatment indicate statistically significant differences as determined by ANOVA (*p* < 0.05).

An overall GRBV acquisition rate of 39% (47/120) was obtained by testing heads with salivary glands of individual *S. festinus* after a 7-day AAP on infected snap bean plants and a 48 h gut clearing period on alfalfa (Table 1). Significantly more specimens from the *S. festinus* SE genotype (72.5%, 29/40) tested positive for GRBV in multiplex PCR compared to the CA genotype (22.5%, 18/80) (*p* = 0.0001, binomial logistic regression; and *p* < 0.0001, Fisher’s exact test), suggesting a greater capacity of the former genotype to acquire GRBV (Table 1). Interestingly, a significantly higher relative GRBV titer was detected in dissected heads with salivary glands of SE individuals in comparison to CA individuals (*p* = 0.0010, Tukey’s Honest Significant Difference) (Figure 3). However, the relative GRBV titer values did not differ significantly by treehopper sex (*p* = 0.0610, Tukey’s Honest Significant Difference) or GRBV isolate (*p* = 0.7367, Tukey’s Honest Significant Difference) (Figure 3).

Across both *S. festinus* genotypes, more individuals acquired GRBV after feeding on snap bean plants infected with the phylogenetic clade II isolate (60%, 24/40) than the phylogenetic clade I isolate (29%, 23/80) but, this difference was not statistically significant (*p* = 0.0669, binomial logistic regression) (Table 1). However, when Fisher’s exact test was performed, the difference was significant (*p* = 0.0014). This is most likely due to effect of the interaction between virus isolate and insect genotype on virus acquisition. Insects of the SE genotype acquired the clade I isolate at a higher rate than the clade II isolate (12/20, 60% vs. 17/20, 85%), but insects of the CA genotype acquired the clade II isolate at a higher rate than the clade I isolate (7/20, 35% vs. 11/60, 21%) (Table 1).

Overall, more females (52.5%, 21/40) than males (32.5%, 26/80) across the two *S. festinus* genotypes acquired GRBV, but this difference was not statistically significant (*p* = 0.9238, binomial logistic regression) (Table 1). However, when Fisher’s exact test was performed, the difference was significant (*p* = 0.0471). Similarly to the interaction described above, this is most likely due to the effect of the interaction between insect sex and genotype on virus acquisition. Males of the SE genotype acquired GRBV at a higher rate than females (16/20, 80% vs. 13/20, 65%), but females of the CA genotype acquired GRBV at a higher rate than males (8/20, 40% vs. 10/60, 17%) (Table 1).

Together, these results revealed differences in the acquisition rate of GRBV in relation to *S. festinus* genotype, sex, and virus isolate. With similar amounts of virus found in infected snap bean plants, *S. festinus* genotype-related difference in virus acquisition can be primarily attributed to GRBV-*S. festinus* interactions or differential feeding behaviors of the two treehopper genotypes or sexes.

### 3.2. Transmission of GRBV by Spissistilus festinus

The transmission rates of GRBV isolates of phylogenetic clades I and II were determined in relation to the genotype (SE and CA) and sex (males only, females only, and an equal number of males and females mixed) of *S. festinus.* Insects underwent a 7-day AAP on infected snap bean plants, followed by a 48 h gut clearing period on alfalfa, and a 14-day IAP on excised snap bean trifoliates before the presence of GRBV in individual trifoliate tissues was assessed by multiplex PCR (Figure 1B). Overall, a 35% (34/96) transmission rate was observed (Table 2).

Individuals of the *S. festinus* SE genotype (46%, 22/48) appeared to be more efficient transmitters of GRBV than individuals of the CA genotype (25%, 12/48), but these differences are not statistically significant (*p* = 0.0679, binomial logistic regression; and *p* = 0.0540, Fisher’s exact test) (Table 2). However, the GRBV isolate of phylogenetic clade II was more frequently transmitted by insects of the SE genotype (54%, 13/24) than by insects of the CA genotype (17%, 4/24) (*p* = 0.0189, binomial logistic regression; and *p* = 0.0146, Fisher’s exact test).

No significant differences in transmission rates were observed between the GRBV isolates of phylogenetic clades I and II (*p* = 0.8278, binomial logistic regression; and *p* = 1, Fisher’s exact test), nor did transmission rates of the two virus isolates differ significantly between the male-only, female-only, and mixed cohorts (*p* = 0.2330, *p* = 0.6327, and *p* = 0.7792, respectively, binomial logistic regression; and *p* = 0.4331, *p* = 1, *p* = 0.7043, respectively, Fisher’s exact test). These results revealed no influence of virus isolate or *S. festinus* sex on the rate of GRBV transmission.

Across the two *S. festinus* genotypes, females (47%, 15/32) transmitted GRBV at a higher rate than males (28%, 9/32) or mixed cohorts (31%, 10/32) (Table 2); however, these differences in transmission rates are not statistically significant (*p* = 0.3884, *p* = 0.9591, and *p* = 0.5789 for male vs. female, male vs. mixed, and female vs. mixed, respectively, binomial logistic regression; and *p* = 0.1963, *p* = 1, *p* = 0.3055, respectively, Fisher’s exact test). Analysis of the interaction between *S. festinus* genotype and sex indicated that transmission rates of GRBV by the male-only and mixed cohorts did not differ significantly between insects of the SE and CA genotypes (*p* = 0.6174 and *p* = 0.7792, respectively, binomial logistic regression; and *p* = 1 and *p =* 1, respectively, Fisher’s exact test). However, GRBV transmission rates by female-only cohorts of *S. festinus* were significantly higher by specimens of the SE genotype (75%, 12/16) than the CA genotype (19%, 3/16) (*p* = 0.0031, binomial logistic regression; and *p* = 0.0038, Fisher’s exact test).

### 3.3. Retention of GRBV by Spissistilus festinus

Retention of GRBV variants of phylogenetic clades I and II was determined in relation to the genotype (SE and CA) and sex (males, females) of *S. festinus* after a 7-day AAP on infected snap bean plants, a 48 h gut clearing period on alfalfa, and subsequent maintenance on alfalfa by testing the presence of the virus in dissected heads with salivary glands from individual treehoppers by multiplex PCR at monthly intervals (T1, T2, T3, and T4) over four months following virus acquisition and gut clearing, which was considered timepoint T0 (Figure 2). Overall, similar rates of GRBV acquisition were obtained for the *S. festinus* SE genotype (87%, 34/39) and the CA genotype (95%, 37/39) at T0 (*p* = 0.9995, binomial logistic regression; and 0.4309, Fisher’s exact test) (Table 3). None of salivary glands (0%, 0/19) or guts (0%, 0/19) of the treehoppers that fed on mock-inoculated plants tested positive for GRBV at T0, as anticipated.

Retention of GRBV by individuals of the two *S. festinus* genotypes was documented up to 90 days post-AAP (Table 3). At 30 days post-AAP (T1), a greater proportion of insects of the *S. festinus* SE genotype (43%, 13/30) than of the CA genotype (33%, 13/40) retained GRBV, but this difference is not statistically significant (*p* = 0.2888, binomial logistic regression; and *p* = 0.4547, Fisher’s exact test). At 60 days post-AAP (T2), no difference in the retention rate of GRBV was observed between the SE genotype (45%, 9/20) and CA genotype (46%, 14/30) (*p* = 0.4941, binomial logistic regression; and *p* = 1, Fisher’s exact test). As expected, none of the salivary glands (0%, 0/19) or guts (0%, 0/20) at 30 days post-AAP and none of the salivary glands (0%, 0/20) or guts (0%, 0/20) at 60 days post-AAP from the treehoppers that fed on mock-inoculated plants tested positive for GRBV.

The overall GRBV retention rate remained similar at 30 (37%, 26/70) and 60 days post-AAP (46%, 23/50) (Table 3), although the number of live insects substantially declined by 25% from T1 to T2 (Supplementary Figure S1). However, by 90 days post-AAP (T3), the overall retention rate dropped substantially, with only a single individual of the *S. festinus* CA genotype (5%, 1/19) and none (0%, 0/3) of the few SE genotype specimens that were still alive (Supplementary Figure S1) retained GRBV (Table 3). As anticipated, none of the salivary glands (0%, 0/3) or guts (0%, 0/3) of the treehoppers that fed on mock-inoculated plants tested positive for GRBV at 90 days. At 120 days post-AAP (T4), none of the SE treehoppers survived (Supplementary Figure S1) and none of the surviving individuals of the CA genotype (0%, 0/21) retained the virus (Table 3). These results demonstrated the capacity of both genotypes of *S. festinus* to retain GRBV isolates of the two phylogenetic clades for at least 60 days post-AAP.

## 4. Discussion

This is the first comprehensive study on the transmission of GRBV by *S. festinus*. By thoroughly exploring acquisition, transmission, and retention dynamics, we addressed knowledge gaps in transmission biology and established a foundation for understanding how *S. festinus* and GRBV diversity may contribute to the epidemiology of red blotch disease. While *S. festinus* has long been suspected to be a vector of GRBV [29] and was more recently recognized as a vector of GRBV [5], little was known about the specific biological factors that govern the efficiency of GRBV transmission. Our study addressed this knowledge deficit by systematically examining GRBV acquisition, transmission, and retention across *S. festinus* genotypes (CA and SE) and sex (male and female), as well as virus isolates of two distinct phylogenetic clades (I and II), by using snap beans as virus donor and virus recipient material under controlled conditions.

Our findings provide insights into GRBV acquisition by *S. festinus* with specimens of the SE genotype displaying a significantly greater capacity for acquisition of GRBV (72.5%, 29/40) than specimens of the CA genotype (22.5%, 18/80) (Table 1), despite having access to similar virus titers in the infected snap bean plants to which they were exposed. These data paired with increased virus titers in the salivary glands of dissected heads of SE individuals compared to CA individuals (Figure 3), suggest the SE genotype has a greater proficiency of GRBV acquisition. One possible explanation of the acquisition differences between the two *S. festinus* genotypes could be distinct feeding behaviors, e.g., feeding in distinct locations on infected plants [7,14]. Another explanation could be a differential efficiency of GRBV transit through the bodies of insects of each genotype to reach the salivary glands due to distinct internal anatomical or biological barriers [30]. Follow-up studies using electrical penetration graphs [31] and detection assays to monitor the virus movement through the treehopper body, such as fluorescence in situ hybridization [32], could help clarify the mechanisms underlying these distinct acquisition rates. Together, our findings underscore the importance of vector characteristics in shaping acquisition dynamics and highlight the complexity of GRBV-*S. festinus* interactions.

The results of our transmission assays revealed notable differences in GRBV transmission rate based on *S. festinus* genotype and virus isolate. Treehoppers of the *S. festinus* SE genotype more efficiently transmitted the GRBV isolate belonging to phylogenetic clade II than those of the CA genotype (Table 2), as initially hypothesized; however, no difference was observed in the transmission frequency of the GRBV isolate belonging to clade I. Similarly, *S. festinus* sex influenced the virus transmission rate, with SE males more likely to acquire GRBV, but SE females significantly more likely to transmit GRBV (Table 2). In contrast, CA males transmitted GRBV more efficiently than CA females, consistent with previous findings [7], although CA females showed higher acquisition rates of GRBV than CA males. It was previously hypothesized that this sex-based difference in the *S. festinus* CA genotype was driven by male-biased movement behaviors [7,8,11]; our results suggest that such treehopper movement may favor acquisition potential, while transmission may depend more on feeding behaviors or internal virus-vector interactions. For example, differences in feeding location on snap bean trifoliates were observed between *S. festinus* SE and CA females during this study. Consistent with previous observations [7], CA females moved lower on the petioles of snap bean trifoliates, whereas SE females were more often found feeding along the petiole-leaf junctions. Another possible explanation for the variability in transmission between GRBV isolates could be due a slight amino acid divergence (1.8%, 4 amino acids of 224) in the coat protein sequence between GRBV isolates of phylogenetic clade I (NY175) and II (NY358) [19]; one or more of these four divergent amino acids may favor or reduce the virus movement from the gut epithelium to the hemolymph and then from the hemolymph to the salivary glands. Interestingly, GRBV variants of phylogenetic clade II are the most prevalent in California vineyards [1,15]. An enhanced acquisition of the phylogenetic clade II isolate by the CA genotype (Table 1) but not transmission (Table 2) may explain the dominance of such isolates in the field. Another explanation may simply be the possible prevalence of GRBV isolates of phylogenetic clade II in the planting material.

*S. festinus* retained GRBV for up to 60 days post-AAP regardless of sex and virus isolate (Table 3). Retention of GRBV in *S. festinus* remained consistent at 30 and 60 days post-AAP, but by 90 days post-AAP, it dropped sharply, with only a single CA genotype individual retaining the virus. GRBV was rarely detected in gut tissue at 60 days post-AAP and was no longer detectable at 90 days post-AAP but remained detectable in the dissected heads with salivary glands at these two time points, consistent with circulative, nonpropagative transmission [5,9]. The ability to retain GRBV for several weeks after acquisition means that even treehoppers, which exhibit low transmission efficiency relative to other geminivirus vectors [9], may still pose a risk of moving the virus long after leaving an infected plant. In vineyard landscapes, this extended infectious period could facilitate virus movement into clean blocks or between cultivated vines and wild *Vitis* hosts in adjacent riparian areas, underscoring the need for management approaches that account for vector movement across habitat boundaries.

Comparisons between the two *S. festinus* genotypes revealed that, while they differed in their acquisition of GRBV, transmission efficiency and virus retention rates were similar. Considering that these genotypes originate from distinct regions, it is possible that virus–vector relationships have evolved and diverged geographically. Specifically, the GRBV clade II isolate was transmitted at higher rates overall, particularly by the CA genotype, whereas the GRBV clade I isolate was more efficiently transmitted by the SE genotype. This pattern may reflect evolutionary divergence in compatibility between virus isolates and vector genotypes, driven by historical patterns of co-occurrence between local vector populations and circulating GRBV clades, such as the previously noted higher prevalence of clade II in California [1,15]. This relationship could explain why differences shaping acquisition were observed in this study without majorly influencing transmission or affecting retention. In addition to virus isolates, the effect of sex was documented on acquisition and transmission, differing between *S. festinus* genotypes. Males of the CA genotype transmitted GRBV more efficiently than females, whereas the opposite trend was observed for the SE genotype. Seasonal dynamics, feeding strategies, and host plant availability can affect development rates, feeding behavior, and reproductive strategies specific to each location, and could in turn influence vector competence. Together, these results highlight that both genotype and sex can influence GRBV transmission, and that regional environmental factors may shape vector life-history traits in ways that affect virus spread. Understanding these interactions is important for predicting transmission dynamics and managing GRBV in different grape-growing regions.

The transmission dynamics observed in this study are consistent with those reported for other circulative, nonpropagative viruses in the family *Geminiviridae* [33,34,35]. However, the genotype- and sex-specific differences we observed suggest that even within a single vector species, transmission potential can vary in ways not fully captured yet. Additional factors not examined in this study that may influence transmission rates include endosymbiont composition, movement behaviors of *S. festinus*, and insect-specific viruses such as a reovirus previously identified in *S. festinus* [36]. These factors may also influence GRBV acquisition, transmission, and retention, as has been shown in other plant virus systems [37]. Numerous layers of biological complexity may explain some of the variability in red blotch disease spread observed in vineyard studies, with inconsistencies observed across space and time [10,15,16,38]. While our work highlights the influence of vector genotype and sex as well as viral diversity on GRBV transmission, understanding how these factors interact with endosymbionts, insect viruses or ecological context will be key to refining disease management strategies.

All experiments in this study were conducted using snap bean as both donor and recipient tissue of GRBV. This tractable, herbaceous system is elegant for monitoring GRBV transmission under controlled conditions. While snap bean supports systemic GRBV infection to some extent and has been widely used in previous transmission assays [5,6,7], it is not a natural host of the virus. Whether similar acquisition, transmission, and retention patterns would occur using grapevine remains an open question. Grapevine tissue differs structurally and chemically from snap bean, which could influence feeding behavior of *S. festinus*, virus titer, or vector interaction with the phloem. In particular, petiole anatomy, tissue toughness, or secondary metabolite profiles could affect the frequency and success of phloem access. While past studies have demonstrated that *S. festinus* transmits GRBV from and to grapevine under experimental conditions [6], direct comparisons of transmission efficiency across host plant species are lacking. It remains possible that some of the genotype- or sex-specific patterns observed here may shift in a grapevine-based system, especially under vineyard conditions.

Taken together, our findings reveal that GRBV transmission by *S. festinus* is shaped by both vector genotype and sex, as well as by virus genotype, with additional biological and ecological factors remaining to be explored. By linking controlled experimental results to patterns observed in vineyards, this work provides a clearer understanding of the mechanisms that underlie red blotch disease spread. These insights strengthen the foundation for developing management strategies that account for variation in vector populations, extended virus retention, and movement between wine grape cultivars and free-living *Vitis* hosts in California and Oregon [6,10,15,16,39,40]. Future studies that test these dynamics in grapevine under vineyard conditions will be critical for translating this knowledge into effective, targeted control measures for GRBV.

## Figures and Tables

**Table 1:** Acquisition rate of grapevine red blotch virus (GRBV) isolates from phylogenetic clades I and II in dissected heads with salivary glands of *Spissistilus festinus* males and females from the Southeast and California genotypes, as determined by multiplex PCR.

	Southeast ^a^			California ^b^			
*S. festinus*/GRBV	Clade I ^c^	Clade II ^d^	Subtotal	Clade I	Clade II	Subtotal	Total
Males	7/10 ^e^	9/10	16/20 (80%)	7/50	3/10	10/60 (17%)	26/80 (32.5%)
Females	5/10	8/10	13/20 (65%)	4/10	4/10	8/20 (40%)	21/40 (52.5%)
Total	12/20 (60%)	17/20 (85%)	29/40 (72.5%)	11/60 (21%)	7/20 (35%)	18/80 (22.5%)	47/120 (39%)

^a^ Southeast: *S. festinus* Southeast genotype; ^b^ California: *S. festinus* California genotype; ^c^ Clade I: GRBV isolate NY175; ^d^ Clade II: GRBV isolate NY358; ^e^ Fractions represent the number of dissected heads with salivary glands that tested positive for GRBV over the total number of dissected heads with salivary glands tested.

**Table 2:** Transmission rates of grapevine red blotch virus (GRBV) isolates from phylogenetic clades I and II by *Spissistilus festinus* males and females from the Southeast and California genotypes, and from mixed cohorts of males and females, as shown by multiplex PCR.

	Southeast ^a^			California ^b^			
*S. festinus*/GRBV	Clade I ^c^	Clade II ^d^	Subtotal	Clade I	Clade II	Subtotal	Total
Males	2/8 ^e^	3/8	5/16 (31%)	1/8	3/8	4/16 (25%)	9/32 (28%)
Females	6/8	6/8	12/16 (75%)	2/8	1/8	3/16 (19%)	15/32 (47%)
Males + Females	1/8	4/8	5/16 (31%)	5/8	0/8	5/16 (31%)	10/32 (31%)
Total	9/24 (38%)	13/24 (54%)	22/48 (46%)	8/24 (33%)	4/24 (17%)	12/48 (25%)	34/96 (35%)

^a^ Southeast: *S. festinus* Southeast genotype; ^b^ California: *S. festinus* California genotype; ^c^ Clade I: GRBV isolate NY175; ^d^ Clade II: GRBV isolate NY358; ^e^ Fractions represent the number of dissected heads with salivary glands that tested positive for GRBV over the total number of dissected heads with salivary glands tested.

**Table 3:** Retention of grapevine red blotch virus (GRBV) isolates from phylogenetic clades I and II by *Spissistilus festinus* males and females from the Southeast and California genotypes, as shown by multiplex PCR.

		T0 (0 dpa) ^c^				T1 (30 dpa)				T2 (60 dpa)				T3 (90 dpa)				T4 (120 dpa)			
		Clade I ^d^		Clade II ^e^		Clade I		Clade II		Clade I		Clade II		Clade I		Clade II		Clade I		Clade II	
*S. festinus*		SG ^f^	G ^g^	SG	G	SG	G	SG	G	SG	G	SG	G	SG	G	SG	G	SG	G	SG	G
SE ^a^	Males	8/9 ^h^	5/9	9/10	3/10	4/10	1/10	2/5	0/5	1/5	0/5	3/5	0/5	nt	nt	0/1	0/1	nt	nt	nt	nt
	Females	9/10	4/10	8/10	1/10	4/10	1/10	3/5	1/5	3/5	1/5	2/5	0/5	0/1	0/1	0/1	0/1	nt	nt	nt	nt
	Subtotal	17/19		17/20		8/20		5/10		4/10		5/10		0/1		0/2		nt		nt	
	Total	34/39 (87%)				13/30 (43%)				9/20 (45%)				0/3 (0%)				na			
CA ^b^	Males	9/9	2/9	10/10	4/10	3/10	3/10	4/10	0/10	2/10	1/10	4/10	0/10	0/1	0/1	0/4	0/4	0/10	0/10	0/10	0/10
	Females	9/10	2/10	9/10	3/10	2/10	2/10	4/10	1/10	4/10	0/10	4/10	nt	1/9	0/9	0/6	0/6	0/10	0/10	0/1	0/1
	Subtotal	18/19		19/20		5/20		8/20		6/20		8/10		1/9		0/10		0/10		0/1	
	Total	37/39 (95%)				13/40 (32.5%)				14/30 (47%)				1/19 (5%)				0/11 (0%)			

^a^ SE: *S. festinus* Southeast genotype; ^b^ CA: *S. festinus* California genotype; ^c^ dpa: days post-acquisition and gut clearing of GRBV; ^d^ Clade I: GRBV isolate NY175; ^e^ Clade II: GRBV isolate NY358; ^f^ SG: Heads with salivary glands; ^g^ G: Guts; ^h^ Fractions represent the number of *S. festinus* specimens that tested positive for GRBV over the total number of specimens tested; nt: not tested; na: not applicable.

## Data Availability

Raw data will be made available upon request.

## Supplementary Materials

The following supporting information can be downloaded at: https://www.mdpi.com/article/10.3390/v17091274/s1, Supplementary Figure S1: Mortality of *Spissistilus festinus* over the course of the grapevine red blotch virus (GRBV) retention experiment.

## Abbreviations

The following abbreviations are used in this manuscript:

AAP	acquisition access period
CA	California *S. festinus* genotype
dpa	days post-acquisition
G	gut
GRBV	grapevine red blotch virus
IAP	inoculation access period
ORF	open reading frame
PCR	polymerase chain reaction
qPCR	quantitative PCR
RCA	rolling circle amplification
SE	southeastern *S. festinus* genotype
SG	heads with salivary glands
