# Supplementary material for: Comparative Acquisition, Transmission, and Retention of Distinct Grapevine Red Blotch Virus Isolates in Relation to the Genotype and Sex of Spissistilus festinus, the Treehopper Vector
Source: Viruses. 2025 Sep 20;17(9):1274. doi: 10.3390/v17091274 (PMC12474397; doi:10.3390/v17091274)
Supplement: Supplementary file 1 [file viruses-17-01274-s001.zip › viruses-3867908-supplementary.pdf]

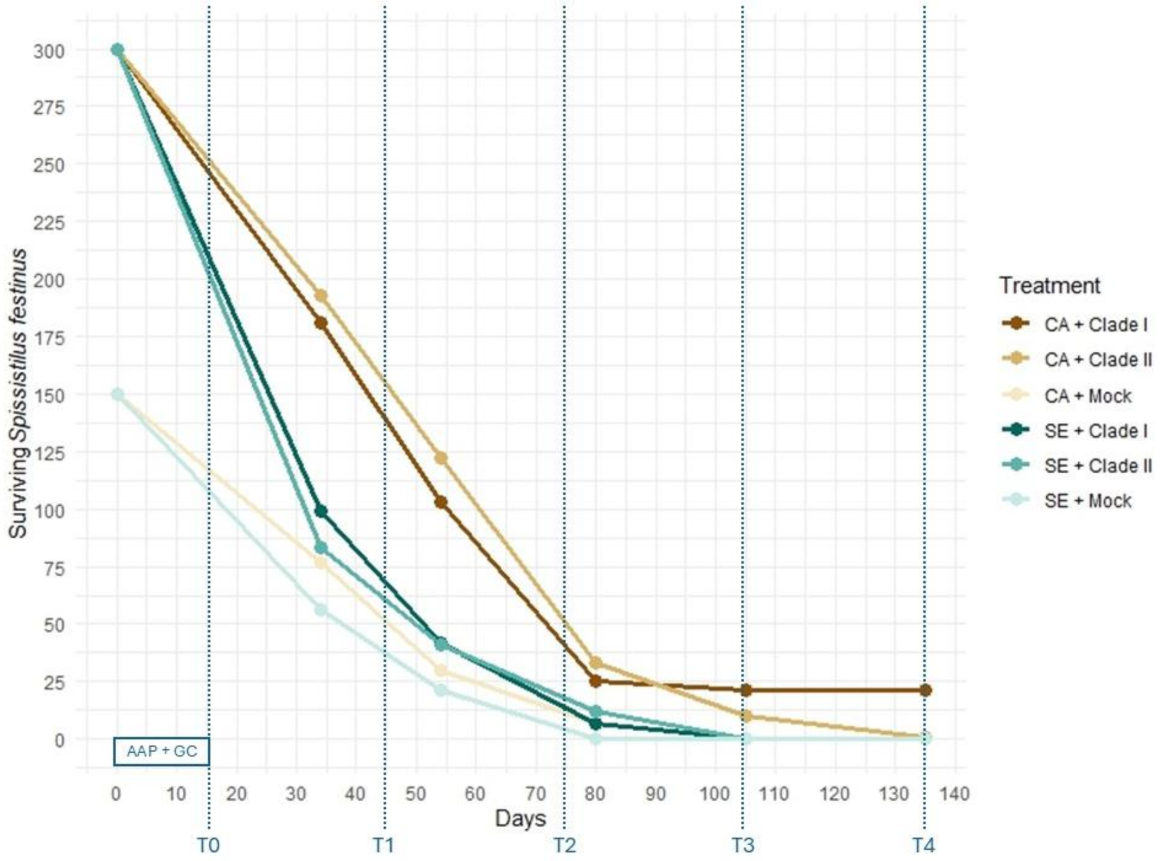

Supplementary Figure S1. Mortality of *Spissistilus festinus* over the course of the grapevine red blotch virus (GRBV) retention experiment. Treatments included populations of either the California (CA) or Southeast (SE) genotypes of *S. festinus* allowed to feed on snap bean (*Phaseolus vulgaris)* plants pinprick-inoculated with GRBV isolate NY175 (phylogenetic clade I), GRBV isolate NY358 (phylogenetic clade II), or mock inoculated (Mock), for a 14-day acquisition access period (AAP) followed by a 48-hour gut clearing period (GC) period on alfalfa (*Medicago sativa*). Following the AAP and GC, insects were maintained on alfalfa. A subset of 20 insects from virus-inoculated treatments or 10 insects from mock-inoculated treatments was collected from each treatment at each experimental timepoint (T0, T1, T2, T3, and T4; or 0, 30, 60, 90, and 120 days after the GC, respectively), until population numbers were too low to permit sample collection.
